# Efficiency of aerobic biodegradation of sugar beet distillery stillage under dissolved oxygen tension-controlled conditions

**DOI:** 10.1371/journal.pone.0306330

**Published:** 2024-07-05

**Authors:** Krzysztof Lutosławski, Agnieszka Ryznar-Luty, Edmund Cibis

**Affiliations:** 1 Department of Process Management, Faculty of Business and Management, Wroclaw University of Economics and Business, Wrocław, Poland; 2 Department of Bioprocess Engineering, Faculty of Production Engineering, Wroclaw University of Economics and Business, Wrocław, Poland; Shanghai Jiao Tong University, CHINA

## Abstract

The efficiency of aerobic biodegradation of distillery wastewater using various microbial cultures is intricately linked to process conditions. The study aimed to examine the aerobic biodegradation by a *Bacillus* bacteria under controlled dissolved oxygen tension (DOT) conditions as a novel approach in the treatment of sugar beet distillery stillage. The processes were conducted in a 2-L Biostat^®^B stirred-tank reactor (STR), at a temperature of 36°C, with aeration of 1.0 L/(L·min), and uncontrolled pH of the medium (an initial pH of 8.0). Each experiment was performed at a different DOT setpoint: 75%, 65% and 55% saturation, controlled through stirrer rotational speed adjustments. The study showed that the DOT setpoint did not influence the process efficiency, determined by the pollutant load removal expressed as COD, BOD_5_ and TOC. In all three experiments, the obtained reduction values of these parameters were comparable, falling within the narrow ranges of 78.6–78.7%, 97.3–98.0% and 75.0–76.4%, respectively. However, the DOT setpoint did influence the rate of process biodegradation. The removal rate of the pollutant load expressed as COD, was the lowest when DOT was set at 55% (0.48 g O_2_/(L•h)), and the highest when DOT was set at 65% (0.55 g O_2_/(L•h)). For biogenic elements (nitrogen and phosphorus), a beneficial effect was observed at a low setpoint of controlled DOT during biodegradation. The maximum extent of removal of both total nitrogen (54%) and total phosphorus (67.8%) was achieved at the lowest DOT setpoint (55%). The findings suggest that conducting the batch aerobic process biodegradation of sugar beet stillage at a relatively low DOT setpoint in the medium might achieve high efficiency pollutant load removal and potentially lead to a reduction in the process cost.

## Introduction

Increasing ecological awareness and stringent environmental regulations have underscored the importance of addressing problems associated with industrial wastewater management and treatment, including from the agri-food sector, which has become an area of high priority in research. The pursuit of a circular economy has prompted efforts towards innovating methods for bioconverting waste into high-value products [1–3]. Agri-food effluent is typically characterised by a high content of organic substances that are difficult to biodegrade, as well as large amounts of inorganic compounds, notably nitrogen and phosphorus [4]. Thus, the development and implementation of highly efficient and cost-effective modern wastewater management technologies play a key role in mitigating the adverse impact of such effluent on the natural environment [5]. Consequently, alternative approaches to bioconversion for liquid waste requiring purification are being explored, enabling the pre-treatment of wastewater for subsequent release into sewage systems and transportation to sewage treatment plants. Numerous studies have investigated various strategies for managing high-load effluents, including physiochemical [4–6], biological [7] and hybrid methodologies [8].

Stillage, a high-load wastewater generated during the ethanol production process from both starch and sugar raw materials, has a detrimental impact on the environment [9–13]. Stillage is characterised by a dark colour, a low pH and a high initial temperature. Its pollutant load is expressed as COD reaching up to 120 g/L and BOD up to 60 g/L. This distillery effluent also contains a high level of inorganic compounds such as nitrogen, potassium, calcium, phosphates and sulphates. The presence of melanoids, with their antioxidant activity, renders them toxic to many microorganisms [12]. Additionally, the high volume of distillery stillage (10 to 15 litres) per 1 litre of ethanol, along with projections for global bioethanol production (137 billion litres by 2026), poses significant environmental concerns [14]. However, existing methods fall short of being able to manage this by-product in an environmentally safe manner.

An effective method for processing distillery stillage is subjecting it to aerobic biodegradation using mixed bacterial cultures. Research to date has shown that in conditions of continuous intensive oxygenation, high efficiency can be achieved for such processes. The achievable extent of pollutant removal exceeds an 88% reduction in the COD value, both for starch and sugar stillage [15–20], while the BOD_5_ can be reduced by as much as 99.4% [15]. However, a key factor affecting the efficiency of this process is the dissolved oxygen tension (DOT) level in the biodegraded medium. Importantly, the highest rate of pollution load reduction is observed in the phase of intensive microflora growth, i.e. at the time when the demand for oxygen by microorganisms is the highest. At this time, the DOT level often drops to zero [15, 17, 18], requiring oxygenation intensification in the medium through the high stirrer rotational speed in bioreactors or special oxygenation systems. Nevertheless, these approaches increase the cost of the process. The cost of aeration can be reduced by using suitably designed bioreactors. One method that can also be used is to maintain DOT at a setpoint by adjusting the stirrer rotational speed in the bioreactor. This system ensures a high supply of oxygen only when it is most needed during the biodegradation. As far as the authors are aware, there are no studies on aerobic biodegradation of distillery stillage under controlled DOT conditions. Due to the above, promising research results on the aerobic treatment of distillery stillage were continued in analogical processes conducted under controlled DOT at relatively low setpoints.

The present study aimed to examine the aerobic biodegradation by a *Bacillus* bacteria under controlled dissolved oxygen tension conditions as a novel approach in the treatment of sugar beet distillery stillage.

## Materials and methods

### Distillery stillage

A batch of beet distillery stillage sourced from the company Rol-Mi-Go Ltd., located in Jaśkowice Legnickie was subject to aerobic biodegradation tests. Prior to testing, the stillage was filtered using filter paper (size 450 x 560 mm, grammage 65 g/m^2^, Chempur, Poland), followed by boiling for 15 minutes to eliminate any possible contamination (the loss of liquid due to boiling was compensated with distilled water). The chemical characterization of the stillage used in biodegradation experiments is presented in Table 1. The pH value of the stillage, resulting from the research programme, was adjusted using 33% NaOH.

**Table 1:** Chemical composition of sugar beet distillery stillage [21] treated in aerobic biodegradation.

Parameter^a^	Value
pH	5.25 ± 0.01
Density (°Blg)	5.50 ± 0.06
SS (suspended solids) (g/L)	1.76 ± 0.08
COD (chemical oxygen demand) (g O_2_/L)	48.30 ± 0.42
COD_sum_^b^ (g O_2_/L)	50.10 ± 0.47
BOD_5_ (five-day biological oxygen demand) (g O_2_/L)	15.50 ± 0.60
TOC (total organic carbon) (g/L)	13.02 ± 0.54
Reducing substances after hydrolysis (g/L)	14.29 ± 0.20
Reducing substances before hydrolysis (g/L)	5.38 ± 0.05
SOA (sum of organic acids) (g/L)	12.06 ± 0.41
Lactic acid (g/L)	4.49 ± 0.23
Glycolic acid (g/L)	2.52 ± 0.13
Acetic acid (g/L)	1.29 ± 0.07
Pyroglutamic acid (g/L)	1.12 ± 0.05
Isobutyric acid (g/L)	0.92 ± 0.06
Butyric acid (g/L)	0.83 ± 0.03
Formic acid (g/L)	0.82 ± 0.04
Propionic acid (g/L)	0.050 ± 0.002
Glycerol (g/L)	3.08 ± 0.06
Betaine (g/L)	0.86 ± 0.06
Total nitrogen (g/L)	1.512 ± 0.030
Ammonia nitrogen (g/L)	0.154 ± 0.009
Total phosphorus (g/L)	0.290 ± 0.004
Phosphate phosphorus (g/L)	0.199 ± 0.002

Results are expressed as the mean of three determinations ± standard deviation of the mean.

^a^Besides pH, density and suspended solids, the parameters were determined after suspended solid separation.

^b^COD determined by the dichromate method + the theoretical COD of betaine.

### Bacterial culture

The biodegradation process of stillage used a mixed culture of thermo- and mesophilic *Bacillus* bacteria isolated from a specific food industry waste processing plant in the United Kingdom. This culture comprised two strains of *B*. *circulans*, and one strain each of *B*. *laterosporus*, *B*. *filicolonicus*, *B*. *stearothermophilus*, *B*. *acidocaldarius*, and *B*. *licheniformis*. The bacterial identification was conducted in previous studies. The conditions for sustaining bacterial activity were delineated in the work by Lutosławski et al. [21].

### Biodegradation processes

Batch biodegradation processes were conducted for 168 hours in a Biostat^®^B STR (stirred-tank reactor) bioreactor with a working capacity of 2 L, equipped with a mixing system manufactured by B. Braun Biotech International. The fermenter was loaded with 1920 mL of distillery stillage and 80 mL of inoculum sourced from an aerated bioreactor without a mixing system. The experiments were conducted at a temperature of 36°C, aeration of 1.0 L/(L·min), without pH regulation (an initial pH value of 8.0), and the initially regulated stirrer rotational speed set at 900 r/min. The initial experimental conditions were determined based on prior research on distillery stillage biodegradation [20, 21]. Each experiment was carried out for different levels of dissolved oxygen tension (DOT setpoint) in the medium, i.e. at 55, 65 and 75% saturation. DOT was maintained at the setpoint value through cascade adjustment of the stirrer rotational speed using a PI type controller installed in the bioreactor. The controller’s parameters were configured as follows: proportional band (Pb) set to 200% and integration time (T_i_) set to 100 s. DOT regulation at the selected level commenced in each process upon the attainment of heightened bacterial activity, immediately following the rapid decrease in DOT concentration in the medium.

### Analytical methods

Bacterial cells were counted using a Thoma chamber (0.05 x 0.05 x 0.1 mm). The content of suspended solids was determined gravimetrically after centrifuging a 60 mL sample at 18.500 *g* for 40 min. in a Sigma^®^4K15 centrifuge. Subsequent analyses were performed on the resulting supernatant. Chemical oxygen demand (COD), five-day biological oxygen demand (BOD_5_), total organic carbon (TOC), total phosphorus, and phosphate phosphorus were measured spectrophotometrically using commercial Hach-Lange cuvette tests (Dr. Bruno Lange GmbH, Macherey-Nagel GmbH). Total nitrogen was assessed using the Kjeldahl method with the C. Gerhardt GmbH & Co. apparatus, whereas ammonia nitrogen with the Parnas apparatus. Organic acids were measured by HPLC (Knauer chromatograph; UV-VIS and RI detectors; Phenomenex ROA column: 7.8 mm i.d. x 300 mm; eluent: 5 mM H_2_SO_4_; flow rate: 0.5 mL/min; temperature: 40ºC). Reducing substances concentration was prepared by a Lane-Eynon method [21]. The concentrations of glycerol and betaine were determined spectrophotometrically. Given that betaine is not detectable via the dichromate method used for COD determination, the COD_sum_ parameter was calculated as the sum of the measured COD and the theoretical COD of betaine (2.097 g O_2_/g of betaine) [21].

The results are reported as the mean value and standard deviation of the mean for n = 3 replicates. Statistical significance (p≤0.05) of the differences between the means of the two groups was verified using an ANOVA test and Duncan’s post hoc test.

## Results and discussion

### Biodegradation efficiency

The effectiveness of sugar beet stillage aerobic biodegradation investigated under dissolved oxygen tension (DOT) control with different setpoints (75%, 65% and 55%) is presented in Table 2. It was found that DOT levels had no significant effect on biodegradation efficiency, specifically pollutant load removal in COD_sum_, BOD_5_ and TOC. Across all experimental conditions, the reduction in these parameter values consistently fell within very narrow ranges: 78.6–78.7%, 97.3–98% and 75.0–76.4%, respectively.

**Table 2:** Biodegradation efficiency of sugar beet distillery stillage under DOT (dissolved oxygen tension) control with different setpoints (75%, 65% and 55% of saturation).

Parameter	DOT control setpoint (%)		
	75	65	55
COD removal (%)	77.6^a^ ± 0.3	77.6^a^ ± 0.4	77.6^a^ ± 0.6
COD_sum_ removal (%)	78.6^a^ ± 0.3	78.7^a^ ± 0.4	78.7^a^ ± 0.5
BOD_5_ removal (%)	97.3^a^ ± 0.4	97.8^a^ ± 0.3	98.0^a^ ± 0.3
TOC removal (%)	75.0^a^ ± 1.2	75.4^a^ ± 1.3	76.4^a^ ± 1.1
Time after which 90% of the overall reduction in COD was attained (h)	55	52.5	59
COD_sum_ removal rate (g O_2_/(L h))	0.52	0.55	0.48
Final number of cells (10^a^/mL)	4.4^a^ ± 0.5	4.0^a^ ± 0.3	3.0 ± 0.4
Final amount of SS formed (g/L)	4.2^a^ ± 0.4	3.9^a^ ± 0.5	3.4^a^ ± 0.5
Y_SS_ ((g final SS formed/g COD_sum_ removed)∙100%)	12.5^a^ ± 1.2	11.2^a^ ± 1.5	10.1^a^ ± 1.4

Results are expressed as the mean ± standard deviation of the mean, n = 3.

^a^Differences in the values denoted by the same letter in the same row are statistically insignificant at p ≤ 0.05.

The efficiency of batch processes with DOT control is comparable to that of an adequate process (operating at 36 °C, with non-regulated pH and aeration speed of 1 L/(L·min)), where DOT is not controlled while maintaining a controlled stirrer rotational speed with a setpoint of 900 r/min. The removal extent of pollutant load expressed by COD_sum_, BOD_5_ and TOC was 79.9%, 98.4% and 76.1%, respectively [21]. As far as the authors of these research results are aware, no references in the literature discuss the aerobic biodegradation of distillery stillage under dissolved oxygen control in the medium. However, regarding other types of wastewater, Kosseva et al. [22] observed a positive effect of holding DOT at specific control levels during the thermophilic degradation of whey using bacteria of the genus *Bacillus*. They achieved the maximum COD reduction (93–94%) in the experiments conducted at 55°C with controlled oxygen content in the medium maintained at the setpoints of 20 and 40%.

However, as reported by several researchers, the provision of intensive oxygenation (mixing and aeration) in the medium is essential for both aerobic thermo- and mesophilic biodegradation of distillery stillage. This requirement is supported by numerous studies investigating the effect of oxygenation conditions on biodegradation efficiency. In batch experiments examining the biodegradation of beet molasses stillage, conducted across shake flask culture (SFC), aerobic bioreactor without mixing (ABWM), and stirred tank reactor with aeration (STR), it was found that the reduction in COD load in SFC and ABWM processes was significantly lower (by at least 37.4%) compared to aerated STR bioreactor experiments [23]. Similarly, Ryznar-Luty et al. [24] demonstrated the lower efficiency of SFC processes compared to STR biodegradation of the same stillage at 58°C, as did Cibis et al. [25] during the purification of potato stillage under mildly thermophilic conditions (45°C). In the processes conducted in an aerated bioreactor, the extent of COD removal was observed to be several to tens of percent higher than in shake flasks. The inefficiency of SFC and ABWM processes was attributed to the low dissolved oxygen content in the substrate medium, resulting from insufficient oxygen input and reduced medium mixing compared to STR bioreactor. This difference in efficiency was evident in continuous biodegradation of starch stillage, where Cibis [26] noted a more than 35% lower COD reduction in STR bioreactor experiments with a hydraulic retention time (HRT) of 40.2 hrs conducted at a constant stirrer rotational speed of 550 r/min compared to 900 r/min. However, a similar correlation between the stirrer rotational speed and biodegradation efficiency was not observed in STR batch processes of beet molasses stillage treatment [24]. Processes conducted at 58°C without pH regulation and at regulated stirrer rotational speeds of 900 r/min and 550 r/min achieved comparable COD reductions, although with a longer HRT of 168 hrs. Therefore, as emphasized in the majority of the cited studies and highlighted by Lasik and Nowak [27], ensuring a sufficient amount of oxygen dissolved in the substrate medium remains a key factor influencing biodegradation efficiency.

The statistical analysis demonstrated that the reduction in pollutant load achieved at a DOT setpoint of 55% is not significantly different from that achieved with a DOT setpoint of 75% (Table 2). This suggests that continuously maintaining a high level of DOT in the medium during the aerobic degradation of distillery sugar beet stillage may not be necessary. Such a revelation holds promise, as it may mitigate the adverse effects of intensive mixing, such as difficulties in medium foaming and biomass separation. Additionally, it may lead to cost reductions in conducting these processes.

**Fig 1:**
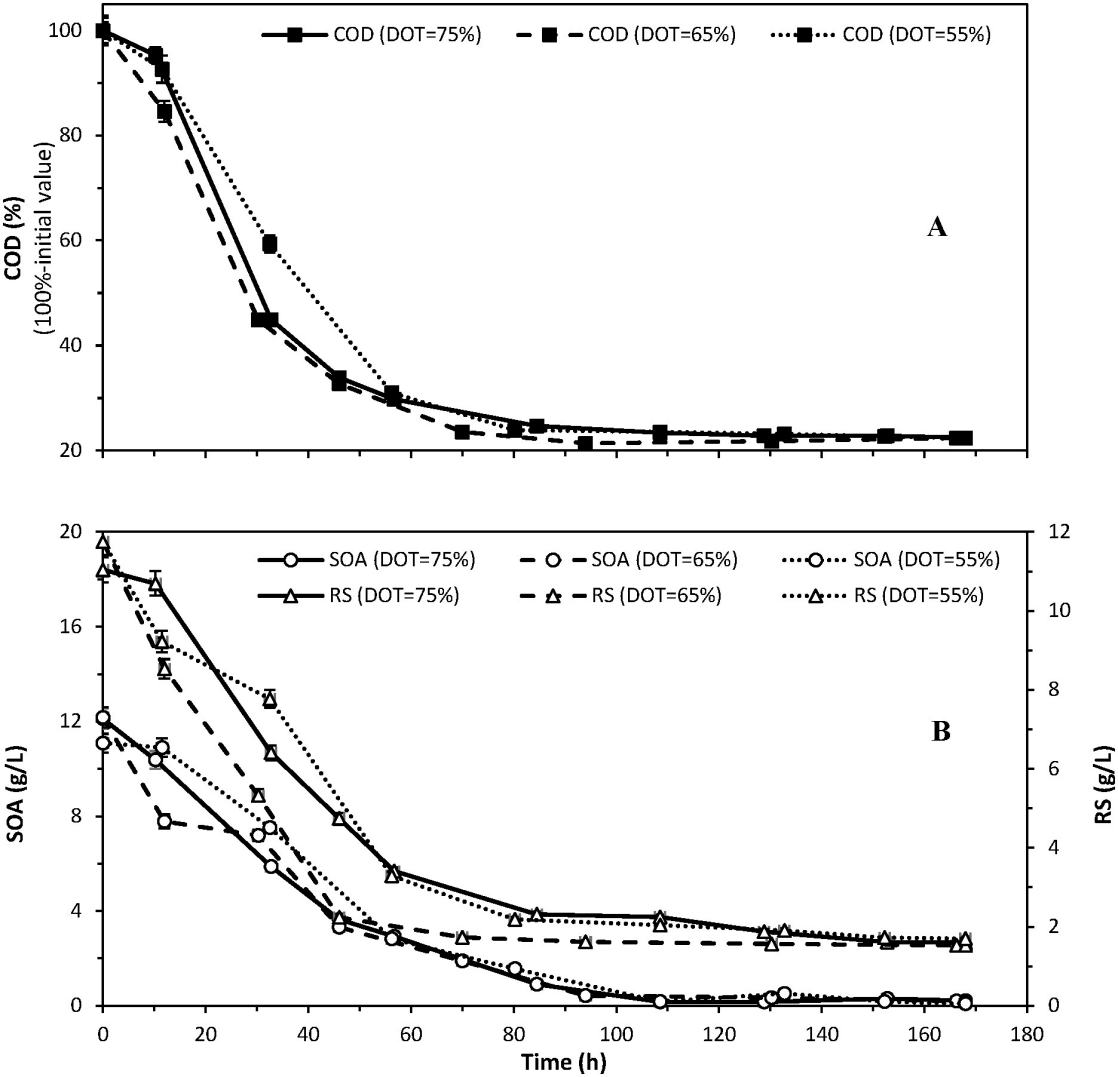
Effect of DOT control with different setpoints (75%, 65% and 55% saturation) on variations in COD (A), reducing substances with hydrolysis (RS) and sum of organic acid (SOA) content (B) during aerobic biodegradation of sugar beet distillery stillage.

Nevertheless, the controlled DOT setpoint significantly influenced the rate of biodegradation processes (Table 2 and Fig 1A). The duration required to achieve a 90% reduction in COD was the longest (59 hrs) in the process with the lowest DOT setpoint (55%), and the shortest (52 hrs)–with a DOT setpoint of 65%. As a result, the latter process achieved the highest COD removal rate (0.55 g O_2_/(L·h)) (Table 2).

It should be underlined that the COD removal rates in all experiments with controlled DOT were higher than in an analogous process with uncontrolled DOT (0.39 g O_2_/(L·h) [21]. Higher COD removal rates were also attained in the biodegradation processes of other distillery effluents with a greater COD pollutant than the sugar beet stillage used when employing the same *Bacillus* bacteria. For instance, Cibis et al. [15] processed beet molasses vinasse at a temperature of 36°C with pH regulated at 8, obtaining a COD_sum_ removal rate of 1.69 g O_2_/(L∙h). Lutosławski et al. [23] noted an even higher value of 2.2 g O_2_/(L∙h) for the same stillage under similar conditions but with a different mixed culture of microorganisms. It should be noted that in the last two cited works, processes without pH regulation yielded considerably lower COD removal rates of 1.17 and 0.9 g O_2_/(L∙h), respectively. Meanwhile, in batch experiments on potato stillage biodegradation conducted at 35°C with pH medium regulation, Krzywonos et al. [16] achieved a COD removal rate of 2.02 g O_2_/(L∙h) using mixed *Bacillus* bacterial cultures. In other food industry sewage biodegradation processes, Kosseva et al. [28] subjected whey to biodegradation at 65°C using *Bacillus* bacteria, achieving a COD removal rate coefficient of 1.57 g O_2_/(L∙h). In contrast, Lasik et al. [29] obtained a maximum COD removal rate of 0.46 g O_2_/(L∙h), lower than that reported in this paper, during batch biodegradation of potato industry wastewater (COD = 35.6 g O_2_/L), using the same mixed *Bacillus* bacterial culture as employed in the case of sugar beet stillage.

### Organic components removal

Lowering the DOT setpoint from 75% to 55% did not result in a decrease in the removal extent of COD_sum_, BOD_5_ and TOC, nor did it decrease the removal extent of the main organic components of the stillage (Table 3). Moreover, operating the process at the lowest DOT setpoint improved the degradation of reducing substances determined before hydrolysis, as well as organic acids (Table 3). Finally, at the tested DOT setpoints, the extent values of removal for reducing substances determined before hydrolysis and after hydrolysis, the sum of organic acids and glycerol, were within relatively narrow ranges (Table 3). In another biodegradation experiment conducted by the authors at a constant stirrer speed of 900 r/min (T = 36°C), the removal extent of both reducing substances and glycerol from beet stillage was lower than that achieved in the process with a controlled DOT at 55%. Specifically, the extent of removal was 81.8% for reducing substances determined after hydrolysis, 83.8% for reducing substances before hydrolysis, and 91.4% for glycerol [21].

**Table 3:** Effect of DOT control with different setpoints (75%, 65% and 55% saturation) on organic components removal during aerobic biodegradation of sugar beet distillery stillage.

Extent of pollutant component removal	DOT control setpoint (%)		
	75	65	55
Reducing substances after hydrolysis (%)	85.5^a^ ± 0.6	87.0 ± 0.5	85.5^a^ ± 0.6
Reducing substances before hydrolysis (%)	79.9 ± 0.6	82.9 ± 0.5	84.2 ± 0.5
Sum of organic acids (%)	98.3 ± 0.1	98.6 ± 0.1	99.2 ± 0.1
Lactic acid (%)	100.0 ± 0.0	100.0 ± 0.0	100.0 ± 0.0
Glycolic acid (%)	97.8^a^ ± 0.2	100.0 ± 0.0	97.8^a^ ± 0.3
Acetic acid (%)	100.0 ± 0.0	100.0 ± 0.0	100.0 ± 0.0
Pyroglutamic acid (%)	100.0 ± 0.0	100.0 ± 0.0	100.0 ± 0.0
Isobutyric acid (%)	97.1 ± 0.3	95.7 ± 0.5	100.0 ± 0.0
Butyric acid (%)	99.1 ± 0.1	100.0 ± 0.0	100.0 ± 0.0
Formic acid (%)	91.1^a^ ± 1.1	90.8^a^ ± 1.1	97.1 ± 0.3
Propionic acid (%)	100.0 ± 0.0	100.0 ± 0.0	100.0 ± 0.0
Glycerol (%)	100.0 ± 0.0	98.2 ± 0.1	100.0 ± 0.0
Betaine (%)	100.0 ± 0.0	100.0 ± 0.0	100.0 ± 0.0
Total nitrogen (%)	50.8^a^^b^ ± 2.9	45.7^a^ ± 3.2	54.0^b^ ± 2.7
Ammonia nitrogen (%)	42.6^a^ ± 6.5	55.7^a^ ± 5.0	52.2^a^ ± 5.4
Total phosphorus (%)	58.4^a^ ± 3.2	58.7^a^ ± 3.2	67.8 ± 2.5
Phosphate phosphorus (%)	39.7^a^ ± 3.0	39.8^a^ ± 3.0	54.1 ± 2.3

Results are expressed as the mean ± standard deviation of the mean, n = 3.

^a, b^Differences in the values denoted by the same letter in the same row are statistically insignificant at p ≤ 0.05.

Regardless of the DOT setpoint, among the organic substances present in the largest amounts in the stillage, bacteria exhibited the fastest assimilation of reducing substances, followed by organic acids (Fig 1B). The slowest assimilation of organic acids was observed at a DOT of 55% (Fig 1B), which undoubtedly affected the slowest COD removal rate (Table 2 and Fig 1A). The same order in the microbial assimilation rate of organic components was also observed during the biodegradation process of beet molasses vinasse carried out at a constant stirrer speed of 900 r/min [21].

The DOT setpoint affected the rate of assimilation of organic acids by the bacteria (Fig 1B), but did not significantly influence their final extent of removal from the medium (Table 3). In the process where the DOT setpoint was the lowest (55%), organic acids were removed from the stillage medium to the highest extent (99.2%). However, this result was only 0.9% higher than the lowest extent (98.3%) achieved in the process with a DOT of 75%, where a slightly lower biodegradation extent of isobutyric, butyric and formic acid was observed than in the process with a DOT of 55% (Table 3).

The slowest biodegradation of organic acids recorded in the process with the lowest DOT setpoint (Fig 1B) was mainly related to the slow absorption by the bacteria of pyroglutamic, formic and propionic acids. Additionally, it is noteworthy that the biodegradation of the sugar beet stillage led to the biosynthesis of lactic and acetic acid, occurring consistently across all three processes (data not presented). Among these acids, lactic acid was synthesised in the highest amounts. Regardless of the process conditions, its concentration after approximately 32 hrs of biodegradation reached a maximum of over 5 times the initial value, up to around 4 g/L (data not presented). Meanwhile, acetic acid was synthesised in smaller amounts, with its maximum concentration in the stillage medium (at 1.8 g/L) observed in the process conducted at DOT = 65%. The lowest amounts of this acid (around 0.3 g/L) were produced in the process conducted with the lowest setpoint of DOT concentration in the medium (55%) (data not presented). As evidenced by the results in Table 2, during the final phase of the biodegradation processes under investigation, the concentration of acetic acid dropped to zero, while the concentration of lactic acid either approached zero or reached it.

In batch processes of aerobic biodegradation of beet molasses vinasse conducted without pH regulation, changes in the concentrations of organic acids were also observed in the degraded medium [19]. Similarly to the findings in this study, these changes were attributed to the simultaneous occurrence of the phenomena of assimilation and biosynthesis of these compounds. Lactic acid production by *Bacillus* bacteria occurred during the phase of intensive growth, often in conditions of oxygen deficiency in the stillage medium. Additionally, valeric acid was produced during the increase in the DOT value. The biosynthesis of acetic and butyric acid was also recorded. In most processes, the synthesised acids were assimilated by the bacteria at later phases of biodegradation [19]. Upon analysing the phenomenon of organic acid biosynthesis in the processes of sugar beet stillage and beet molasses vinasse, as described in the last cited paper, it becomes apparent that this is not dependent on the level of oxygen saturation of the medium.

In the process of whey degradation by bacteria of the genus *Bacillus*, lowering the DOT from 40 to 20% also had no significant effect on the bioconversion rate of both organic acids and lactose. The authors of the referenced study noted that *Bacillus* bacteria exhibit activity over a wide range of oxygen availability under mesophilic as well as thermophilic conditions [30].

### Nitrogen and phosphorus removal

In the process conducted at the lowest dissolved oxygen setpoint (55%), the highest reduction in total nitrogen as well as total phosphorus content in the medium was achieved, reaching 54% and 67.8%, respectively. In the remaining experiments, the extent values of removal of these biogens from the medium did not show statistically significant differences (Table 2). A similar extent of total nitrogen removal (56.4%) was obtained in a batch biodegradation process of the same distillery stillage under continuous intensive oxygenation conditions (900 r/min and 1.0 L/(L·min)). However, the removal efficiency of total phosphorus was nearly 10% lower [21]. Notably, Haddaji et al. [31] reported significantly higher reductions in nitrogen and phosphorus content in refining vegetable oil wastewater. In an aerobic-anoxic sequencing batch reactor, they achieved 93% removal of nitrates, 96% removal of ammonia nitrogen, and 91% removal of phosphates from the biodegraded medium.

**Fig 2:**
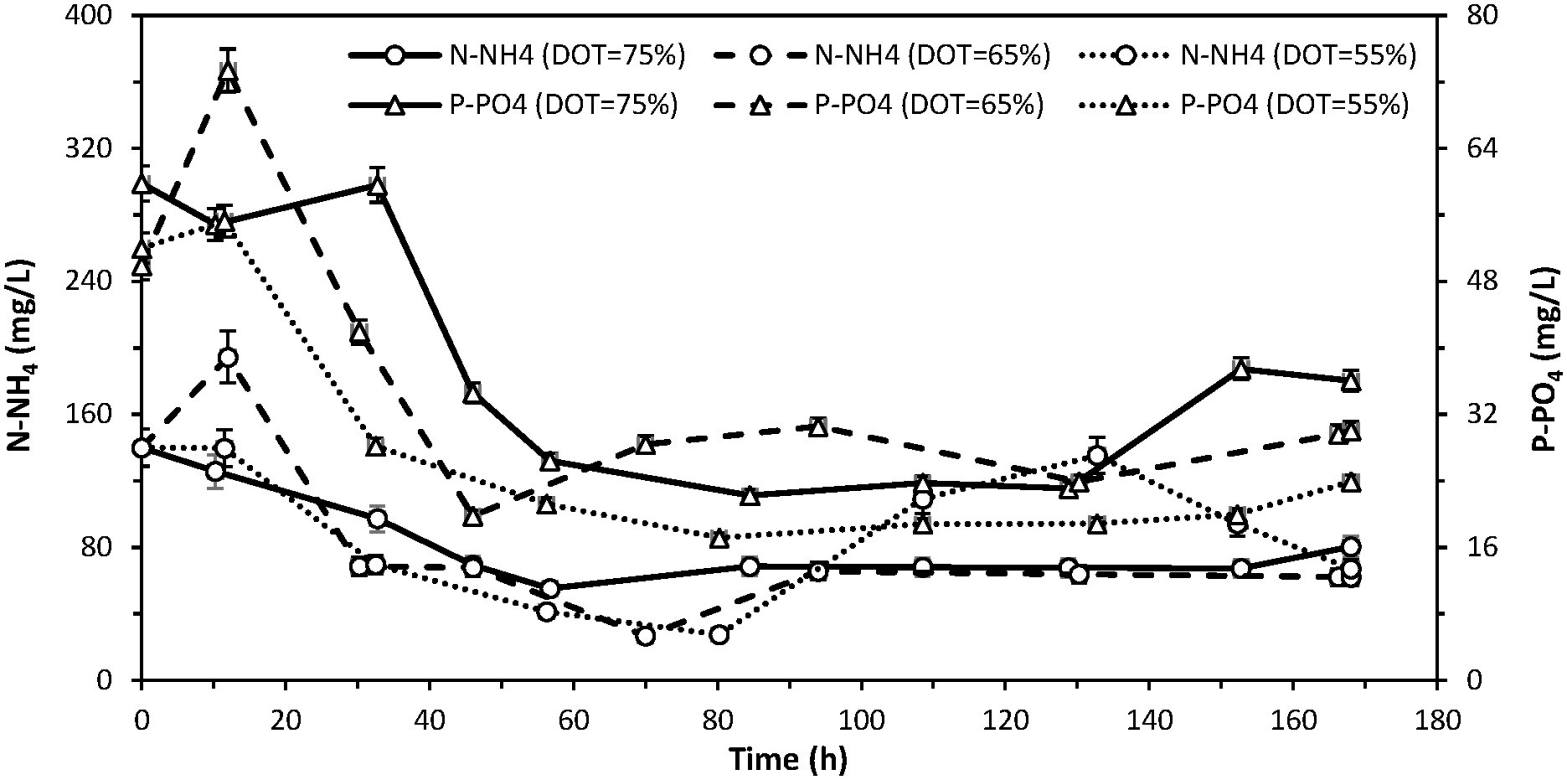
Effect of DOT control with different setpoints (75%, 65% and 55% saturation) on variations in nitrogen and phosphorus content during aerobic biodegradation of sugar beet distillery stillage.

In all the processes, the changes in the content of ammonia nitrogen and phosphate phosphorus followed a similar course (Fig 2). It is worth noting that regardless of the DOT setpoint, no deficiency in the mentioned biogens was observed in the medium during biodegradation. After 12 hrs of biodegradation, coinciding with the commencement of DOT regulation and the sudden increase in biomass observed in every experiment, there was a gradual decrease in both ammonia nitrogen and phosphate phosphorus content in the stillage medium. However, in the process with the highest DOT setpoint, the decrease in P-PO_4_ content occurred later, around 33 hrs into biodegradation. Depending on the adopted DOT setpoint, the minimum concentration of ammonia nitrogen and phosphate phosphorus in the medium was observed between 46 and 85 hrs of biodegradation. The minimum N-NH_4_ content ranged from 27 mg/L (for a DOT of 55% and 65%) to 55 mg/L (for a DOT of 75%), and for P-PO_4_ from 17 mg/L (for a DOT of 55%) to 22 mg/L (for a DOT of 75%). Subsequently, the content of these biogens in the medium slightly increased. It can be noted with some simplification that the increase in their content coincided with a visible drop in biomass content in the medium, as measured by the number of bacteria cells (Figs 2 and 4). A notable increase in phosphate phosphorus content in the degraded stillage was observed towards the end of biodegradation. However, it was evident that in every conducted process, the final concentration of both ammonia nitrogen and phosphate phosphorus in the stillage medium was lower than their respective initial values (Fig 2).

No significant dependencies were observed between the process DOT setpoint and the final content of ammonia nitrogen in the medium. However, in the final hour of biodegradation, the lowest concentration (60 mg/L) was noted when the parameter was regulated at an intermediate level (DOT = 65%) (Fig 2). Conversely, the DOT setpoint exhibited a clear influence on the final phosphate phosphorus content in the medium. A lower level of DOT correlated with a lower final content of this element (ranging from 24 mg/L at DOT = 55% to 36 mg/L at DOT = 75%) (Fig 2), despite observing a smaller final biomass produced at a lower setpoint (Table 2). Finally, the highest removal of P-PO_4_ from the degraded stillage (approximately 54.1%) was achieved at the lowest DOT setpoint. In the remaining experiments (with higher DOT levels), the reduction in P-PO_4_ content in the medium was approximately 14% lower (Table 3).

### DOT, pH and biomass variations

**Fig 3:**
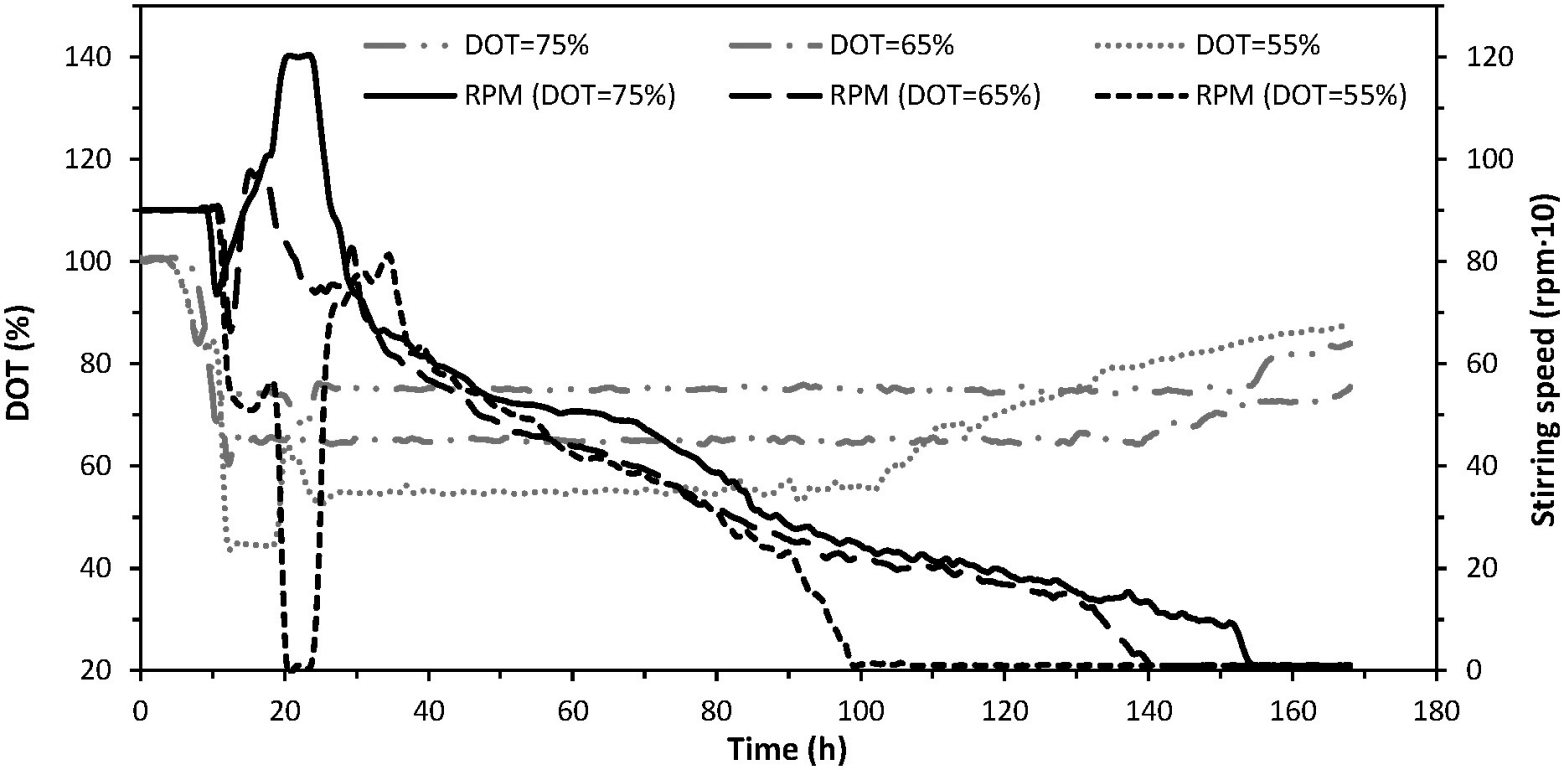
Variations in DOT and stirring speed control signal during aerobic biodegradation of sugar beet distillery stillage under different DOT setpoints (75%, 65% and 55% saturation).

**Fig 4:**
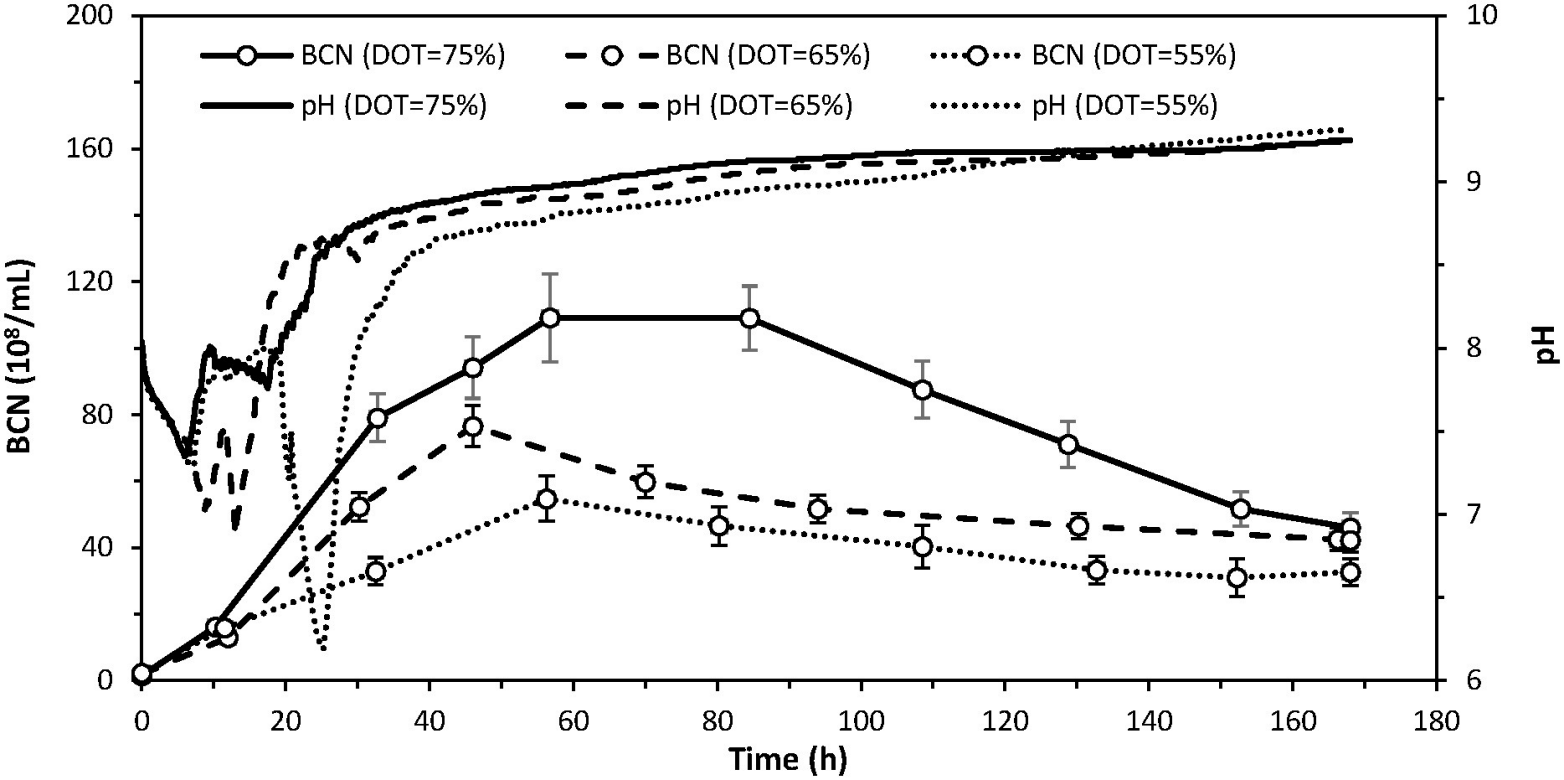
Effect of DOT control with different setpoints (75%, 65% and 55% saturation) on variations in bacterial cell number (BCN) and pH during aerobic biodegradation of sugar beet distillery stillage.

In each conducted process, three further phases of biodegradation can be distinguished. The first phase is a time period necessary for bacterial multiplication and is also the initial stage before DOT control. In this phase (lasting at least 7 hrs), the stirring rotational speed was maintained at a constant level (900 r/min), while the DOT value decreased along with the increase in the number of bacteria cells (Figs 3 and 4).

The following second phase is a time period of intensive biodegradation that begins when the DOT falls below the planned setpoint (thus starting DOT control), continues as the stirrer rotational speed increases to the maximum, and ends when the stirrer rotational speed falls below its maximum value. From the plots presented in Fig 3, concerning the stirrer rotational speed, it can be seen that the biodegradation was most intensive between 15 and 35 hrs. At that time, the highest oxygen demand was recorded in all experiments, leading to an increase in the stirrer rotational speed to the maximum values of 813 r/min (DOT = 55%), 977 r/min (DOT = 65%) and 1204 r/min (DOT = 75%). It should be noted that the oxygen demand in the latter of the mentioned processes exceeded (for several hours) the technical capabilities of the oxygen supply provided by the bioreactor. Furthermore, during the second phase, a decrease in the pH value of the medium was observed to the minimum of 6.2 (DOT = 55%), 6.9 (DOT = 65%) and 7.4 (DOT = 75%) after 25, 13 and 8 hrs, respectively (Fig 4).

The final phase represents a time period characterised by a gradual decrease in the stirrer’s rotational speed in response to the declining oxygen demand within the system (Fig 3). Throughout this phase, the DOT remained mostly stable at the setpoint level. However, as the bacterial activity ceased and the stirrer rotational speed reached zero, the DOT gradually increased above the setpoint (Fig 3). Concurrently, the pH trend in each process exhibited a continuous increase, culminating in a final value of approximately 9.3 at the end of biodegradation (Fig 4). An increase in pH was also observed during the removal of organic load from olive mill wastewater in the biodegradation process by a mixed bacterial culture. It was the result of the bacteria using organic acids as a carbon source [32].

As the biodegradation process finished, despite the stirrer’s rotational speed decreasing almost to zero, there was an observed increase in the DOT content in the medium. At DOT setpoints of 75, 65 and 55%, this phenomenon occurred after 155, 140 and 102 hrs, respectively (Fig 3). Thus, it can be inferred that the heightened oxygen demand during biodegradation with DOT regulation persisted longer as the setpoint of this parameter increased, likely due to the quantity of biomass generated during the process (Fig 4). A sharp increase in biomass content in the medium was observed during a period of high oxygen demand, with its rate of increase accelerating with the higher DOT setpoint (Figs 3 and 4). At DOT = 75%, the highest final number of produced bacterial cells (4.4·10^9^/mL), as well as the highest amount of produced suspended solids (4.2 g/L) were noted (Table 2). Conversely, at DOT = 55%, these parameter values were the lowest, at 3·10^9^/ml and 3.4 g/L, respectively. The lowest final amount of produced suspended solids for a DOT of 55%, coupled with nearly identical reductions in COD_sum_ (78.6–78.7%) across all three processes, resulted in the lowest Y_SS_ parameter value (10.1%) being achieved in the experiment with the lowest DOT setpoint (Table 2).

As previously mentioned, one of the drawbacks of aerobic processes is the necessity for their intensive oxygenation (mixing and aeration). In batch biodegradation processes of sugar beet stillage conducted without DOT regulation, during the phase of intensive growth, the bacterial culture used exhibited a particularly high oxygen demand. This led to a deterioration of oxygen conditions in the bioreactor, resulting in a significant decrease in the dissolved oxygen content in the biodegraded medium, even to values as low as 16.5% [21]. According to the authors of many studies on aerobic biodegradation of wastewater of various origins, including distillery stillage, this phenomenon often leads to an oxygen deficiency (DOT≈0) in the medium [15, 23, 24]. Given that the highest rate of pollutant load removal occurs during the intense absorption of oxygen by microorganisms from the medium, a significant factor determining the high efficiency of the process is ensuring the necessary amount of this element in the biodegraded medium during this period.

## Conclusions

Research indicates that sugar beet distillery stillage biodegradation in a batch system can be equally effective at high as well as low levels of controlled dissolved oxygen tension (DOT). Across various setpoints of this parameter tested, most process efficiency indicators evaluated were at a comparable level, except for process rate. The maximum reductions in COD_sum_ (79%), BOD_5_ (98%) and TOC (76%) were achieved when DOT was maintained at the lowest level (55%). Correspondingly, this condition also resulted in the highest extent of degradation of the main stillage organic components, including reducing substances (85%), organic acids (99%) and glycerol (100%). Moreover, at the lowest DOT setting (55%), the highest removal was observed for total phosphorus (68%) and phosphate phosphorus (54%), with equally high removal for total nitrogen (54%) and ammonia nitrogen (52%), which were not statistically significantly different from those achieved at other DOT levels (75% and 65%).

The study suggests that achieving significant pollutant load reduction in sugar beet stillage using mixed *Bacillus* bacteria culture is feasible even with a relatively low DOT level in the medium, potentially reducing the energy consumption associated with oxygenation. Future research plans involve conducting stillage biodegradation on a semi-technical scale with controlled low DOT levels in both batch and continuous processes. The energy consumption during these processes will be measured to facilitate economic analysis.

The biodegradation method presented in this work can also be used in the utilization of other highly loaded wastewater from the agri-food industry (e.g., from dairies, slaughterhouses, potato or oilseed processing plants), as well as livestock farm effluents. Due to the presence of difficult-to-degrade compounds in many wastewater, aerobic biodegradation may be an element of a comprehensive treatment system. It should be preceded by processes that convert biomass into value-added products in accordance with the biorefinery concept. The parameters of the effluent after biodegradation allow it to be redirected to subsequent treatment stages.
